# Flow cytometric analysis of phosphatidylcholine metabolism using organelle-selective click labeling

**DOI:** 10.1016/j.xpro.2023.102525

**Published:** 2023-08-26

**Authors:** Masaki Tsuchiya, Nobuhiko Tachibana, Itaru Hamachi

**Affiliations:** 1 Department of Synthetic Chemistry and Biological Chemistry, Graduate School of Engineering, Kyoto University, Katsura, Nishikyo-ku, Kyoto 615-8510, Japan; 2 PRESTO (Precursory Research for Embryonic Science and Technology, JST), Sanbancho, Chiyodaku, Tokyo 102-0075, Japan; 3 ERATO (Exploratory Research for Advanced Technology, JST), Sanbancho, Chiyodaku, Tokyo 102-0075, Japan

**Keywords:** Flow Cytometry/Mass Cytometry, Metabolism, Molecular/Chemical Probes, Chemistry

## Abstract

Here, we present a protocol to analyze phosphatidylcholine (PC) metabolism in mammalian cells using organelle-selective click labeling coupled with flow cytometry (O-ClickFC). We describe steps for the metabolic incorporation of azide-choline into PC. We then detail fluorescent labeling of the azide-modified PC with organelle-targeting clickable dyes in the ER-Golgi, plasma membrane, and mitochondria, and by flow cytometry. This protocol is optimized for flow cytometric quantification of the labeled PC at the organelle level within single live cells.

For complete details on the use and execution of this protocol, please refer to Tsuchiya et al. (2023).^1^

## Highlights

• O-ClickFC allows flow cytometric analysis of PC metabolism in live mammalian cells
• High-throughput measurements of PC at the organelle level with single-cell resolution
• A simple and easy-to-use protocol using commercially available reagents

## Abstract (editor highlights)

Publisher’s note: Undertaking any experimental protocol requires adherence to local institutional guidelines for laboratory safety and ethics.

## Graphical abstract

**Figure:**
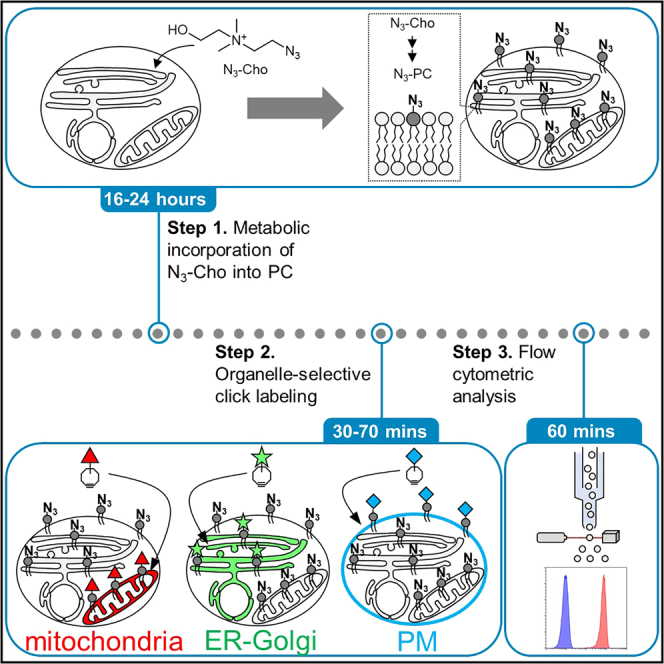

## Before you begin

The protocol presented here describes the basic step-by-step procedures of O-ClickFC^1^ using commercially available resources. This protocol is optimized for K562 human cell line. While the protocol should be applicable for other types of mammalian cells including rodent cells, it may be necessary to optimize the experimental conditions such as the incubation time of N_3_-Cho, in accordance with the cell culture. For flow cytometric analysis, this protocol uses a cell sorter, but should be compatible with a cell analyzer.

## Key resources table

**Table:** 

REAGENT or RESOURCE	SOURCE	IDENTIFIER
1-Azidoethyl-choline	Jena Bioscience	Cat#CLK-065
BDP FL DBCO (BDP-DBCO)	BroadPharm	Cat#BP-23473
DBCO-AF647	Jena Bioscience	Cat#CLK1302-1
Cyanine3 DBCO (Cy3-DBCO)	Lumiprobe	Cat#A10F0
Cyanine5 azide (Cy5-N_3_)	Lumiprobe	Cat#A3030
Mesoxalonitrile 3-chlorophenylhydrazone	FUJIFILM Wako	Cat#034-16993
Valinomycin	FUJIFILM Wako	Cat#223-02391
eBioscience Fixable Viability Dye eFluor 780 (FVD780)	Invitrogen	Cat#65-0865-14

**Experimental models: Cell lines**		

K562	Riken BRC	Cat#RBC0027

**Software and algorithms**		

Cell Sorter software (MA900)	Sony	N/A

**Other**		

IMDM	FUJIFILM Wako	Cat#098-06465
Choline-free IMDM (custom made: choline chloride is removed from the original IMDM medium (FUJIFILM Wako, Cat#098–06465))	Gmep	N/A
High K^+^ RPMI 1640 (custom made: NaCl is replaced by equivalent molar concentration of KCl from the original RPMI 1640 medium (FUJIFILM Wako, Cat#189–02025))	Gmep	N/A
Fetal bovine serum	Sigma-Aldrich	Cat#F7524
Dialyzed fetal bovine serum	Gibco	Cat#26400044
Penicillin-Streptomycin solution, 100×	FUJIFILM Wako	Cat#168-23191

## Materials and equipment

**Table:** Culturing medium

Reagent	Final concentration	Volume (mL)
IMDM		500
FBS	10%	50
Penicillin-Streptomycin Solution, 100×	1×	5
**Total**		555

**Table:** Choline-free medium

Reagent	Final concentration	Volume (mL)
Choline-free IMDM		500
Dialyzed FBS	10%	50
Penicillin-Streptomycin Solution, 100×	1×	5
**Total**		555

**Table:** High K^+^ washing medium (stock)

Reagent	Final concentration	Volume (mL)
High K^+^ RPMI 1640		500
FBS	10%	50
Penicillin-Streptomycin Solution, 100×	1×	5
**Total**		555

***Note:*** All media are stored at 4°C up to 2 months.

**Table:** High K^+^ washing medium (working)

Reagent	Final concentration	Volume (mL)
High K^+^ Washing Medium (Stock)		10
Valinomycin, 20 mM DMSO stock	20 μM	0.01
Mesoxalonitrile 3-chlorophenylhydrazone, 50 mM DMSO stock	50 μM	0.01
**Total**		10

***Note:*** High K^+^ washing medium (working) should be prepared fresh during the procedure and stored at 37°C until use.

### Reagents for click labeling

• N_3_-Cho stock solution: Dissolve appropriate amount of N_3_-Cho with 1× PBS to 1 M.

***Note:*** N_3_-Cho solution is stored at −20°C for short term storage up to 36 months, or −80°C for long term storage.

• BDP-DBCO stock solution: Dissolve BDP-FL-DBCO with DMSO to 10 mM.
• BDP-DBCO working solution: Dilute the stock solution with 4% FBS/IMDM to 100 nM.
• DBCO-AF647 stock solution: Dissolve DBCO-AF647 with DMSO to 10 mM.
• DBCO-AF647 working solution: Dilute the stock solution with 4% FBS/IMDM to 100 μM.
• Cy3-DBCO stock solution: Dissolve Cy3-DBCO with DMSO to 10 mM.
• Cy3-DBCO working solution: Dilute the stock solution with 4% FBS/IMDM to 50 nM.
• Cy5-N_3_ stock solution: Dissolve Cy5-N_3_ with DMSO to 10 mM.
• Cy5-N_3_ stock solution: Dilute the stock solution with 4% FBS/IMDM to 100 nM.

***Note:*** All stock solutions listed above are stored at −20°C for short term storage up to 12 months, or −80°C for long term storage up to 36 months. Avoid repeat freeze/thaw cycle. All working solutions should be prepared fresh during the procedure.

## Step-by-step method details

### Metabolic incorporation of N_3_-Cho

**Timing: 1 h**

**Figure 1:**
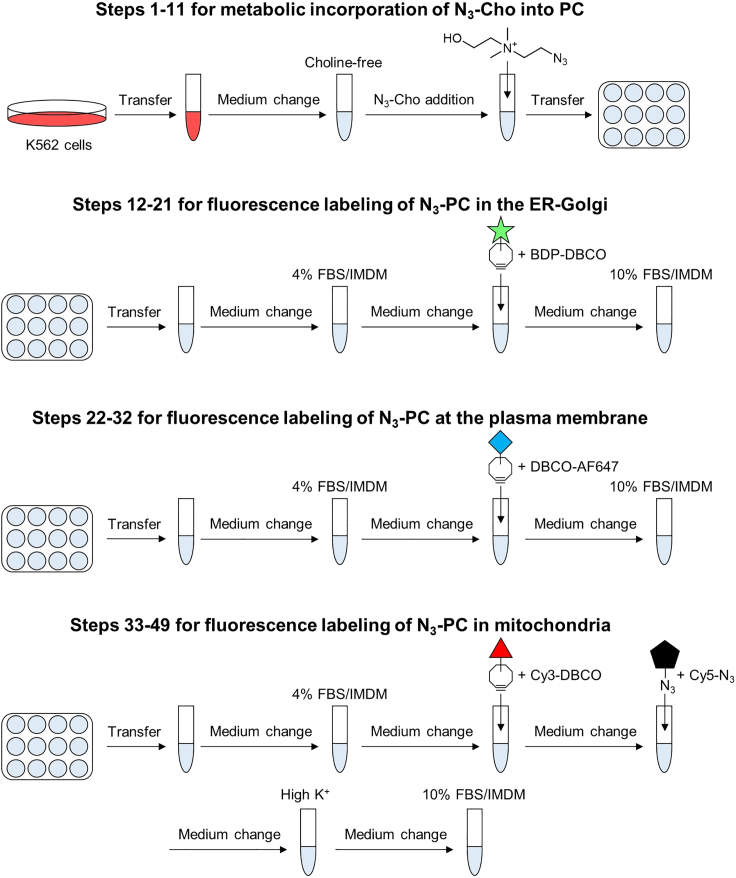
Scheme for metabolic N_3_-Cho incorporation and fluorescent PC labeling Steps 1–11 for metabolic incorporation of N_3_-Cho into PC in K562 cells. Steps 12–21 for PC labeling in ER-Golgi using BDP-DBCO. Steps 22–32 for PC labeling in the plasma membrane using DBCO-AF647. Steps 33–49 for PC labeling in mitochondria using Cy3-DBCO.

This section describes the metabolic incorporation of N_3_-Cho into PC in the K562 cells (Figure 1).

***Note:*** All steps are performed at room temperature otherwise indicated.

1. Maintain K562 cells in the culturing medium in a 10-cm dish in humidified incubator at 37°C, 5% CO_2_.
2. Transfer the cells into 15 mL conical tube.
3. Centrifuge at 500 × *g*, 3 min, and discard supernatant.
4. Resuspend with 5 mL of Cho-free medium.
5. Repeat step 3–4 once again.
6. Resuspend cells with 1 mL of Cho-free medium.

**CRITICAL:** Wash the cells with choline-free medium 2–3 times to completely remove choline residues.

7. Cell count with automated cell counter.
8. Adjust cell density to 0.5–1.0 × 10^6^ cells/mL with Cho-free medium.
9. Add 10 mM N_3_-Cho to final concentration of 10 μM. Mix well.
10. Inoculate the cell suspension into 12-well plate (1 mL/well).
11. Culture cells in humidified incubator at 37°C, 5% CO_2_ for 16–24 h.

***Note:*** At this point, the protocols diverge depending on the target of the interest (ER-Golgi, Plasma membrane or mitochondria). Choose one of the methods below to continue the protocol at step 12 for ER-Golgi, step 22 for plasma membrane, or step 33 for mitochondria.

### Organelle-specific labeling of N_3_-PC

#### Labeling of N_3_-PC in the ER-Golgi

**Timing: 45 min**

This section describes the fluorescence labeling of N_3_-PC using the ER-Golgi-targeting clickable dye **BDP-DBCO** (Figure 1).

***Note:*** 12–21 steps are performed at room temperature.

12. Transfer 200 μL of the cell suspension into 1.5 mL tube.
13. Centrifuge at 1000 × *g*, 1 min. Discard the supernatant.
14. Resuspend with 500 μL of 4% FBS/IMDM.
15. Repeat step 13–14 again.
16. Centrifuge at 1000 × *g*, 1 min. Discard the supernatant.
17. Resuspend cells with 500 μL of **BDP-DBCO working solution.** Mix well by pipetting 3–4 times, then incubate at room temperature for 15 min.

***Optional:*** 1 μL of FVD780 can be added to assess cell viability.

**CRITICAL:** It is important to make cells dispersed during click chemistry reaction.

18. Centrifuge at 1000 × *g* for 1 min, discard the supernatant, and resuspend with 1 mL of 10% FBS/IMDM.
19. Repeat step 18 once more.
20. Resuspend the cells with 500 μL of 10% FBS/IMDM.
21. Filter the cells through 40 μm nylon cell strainer into 5 mL polystyrene tube.

***Note:*** Fluorophore used in this study is relatively stable, and thus filtered cells can be stored at room temperature for up to 8 h without reducing the fluorescence signals. However, it is recommended to analyze the cells as quickly as possible.

(Continued to step 50).

#### Labeling of N_3_-PC at the plasma membrane

**Timing: 1 h**

This section describes the fluorescence labeling of N_3_-PC using the plasma membrane-targeting clickable dye **DBCO-AF647** (Figure 1).

***Note:*** 22–27 steps are performed at 15°C. 28–32 steps are performed at 4°C or on ice.

22. Transfer 200 μL of the cell suspension into 1.5 mL tube.
23. Centrifuge at 1000 × *g*, 1 min. Discard the supernatant.
24. Resuspend with 500 μL of 4% FBS/IMDM.
25. Repeat step 23–24 again.
26. Centrifuge at 1000 × *g*, 1 min. Discard the supernatant.
27. Resuspend cells with 100 μL of **DBCO-AF647 working solution.** Mix well by pipetting 3–4 times, then incubate at 15°C for 30 min.

***Optional:*** 1 μL of FVD780 can be added to assess cell viability.

**CRITICAL:** It is important to make cells dispersed during click chemistry reaction.

28. Centrifuge at 1000 × *g* for 1 min, discard the supernatant, and resuspend with 1 mL of ice-cold 10% FBS/IMDM.
29. Repeat step 28 once more.
30. Centrifuge at 1000 × *g* for 1 min. Discard the supernatant
31. Resuspend with 500 μL of ice-cold 10% FBS/IMDM.
32. Filter the cells through 40 μm nylon cell strainer into 5 mL polystyrene tube.

***Note:*** Fluorophore used in this study is relatively stable, and thus filtered cells can be stored at room temperature for up to 8 h without reducing the fluorescence signals. However, it is recommended to analyze the cells as quickly as possible.

(Continued to step 50).

#### Labeling of N_3_-PC in mitochondria

**Timing: 2 h**

This section describes the fluorescence labeling of N_3_-PC using the mitochondria-targeting clickable dye **Cy3-DBCO** (Figure 1).

***Note:*** Steps 38, 40, 42, and 45 are performed at 37°C. The other steps are performed at room temperature.

33. Transfer 200 μL of the cell suspension into 1.5 mL tube.
34. Centrifuge at 1000 × *g*, 1 min. Discard the supernatant.
35. Resuspend with 500 μL of 4% FBS/IMDM.
36. Repeat step 34–35 again.
37. Centrifuge at 1000 × *g*, 1 min. Discard the supernatant.
38. Resuspend cells with 200 μL of warm **Cy3-DBCO working solution.** Mix well by pipetting 3–4 times, then incubate at 37°C for 15 min.

***Optional:*** 1 μL of FVD780 can be added to assess cell viability.

**CRITICAL:** It is important to make cells dispersed during click chemistry reaction.

39. Centrifuge at 1000 × *g* for 1 min. Discard supernatant.
40. Resuspend cells with 200 μL of warm **Cy5-N3 working solution.** Mix well by pipetting 3–4 times, then incubate at 37°C for 15 min.
41. Centrifuge at 1000 × *g* for 1 min. Discard supernatant.
42. Resuspend cells with 500 μL of warm High K + Washing Medium (Working). Mix well by pipetting 3–4 times, then incubate at 37°C for 15 min.
43. Repeat Steps 41–42 twice more.
44. Centrifuge at 1000 × *g* for 1 min. Discard supernatant.
45. Resuspend cells with 500 μL of warm 10% FBS/IMDM. Mix well by pipetting 3–4 times, then incubate at 37°C for 15 min.
46. Repeat Steps 44–45 twice more.
47. Centrifuge at 1000 × *g* for 1 min. Discard supernatant.
48. Resuspend the cells with 500 μL of 10% FBS/IMDM.
49. Filter the cells through 40 μm nylon cell strainer into 5 mL polystyrene tube.

***Note:*** Fluorophore used in this study is relatively stable, and thus filtered cells can be stored at room temperature for up to 8 h without reducing the fluorescence signals. However, it is recommended to analyze the cells as quickly as possible.

### Flow cytometry

**Timing: 1 h**

**Figure 2:**
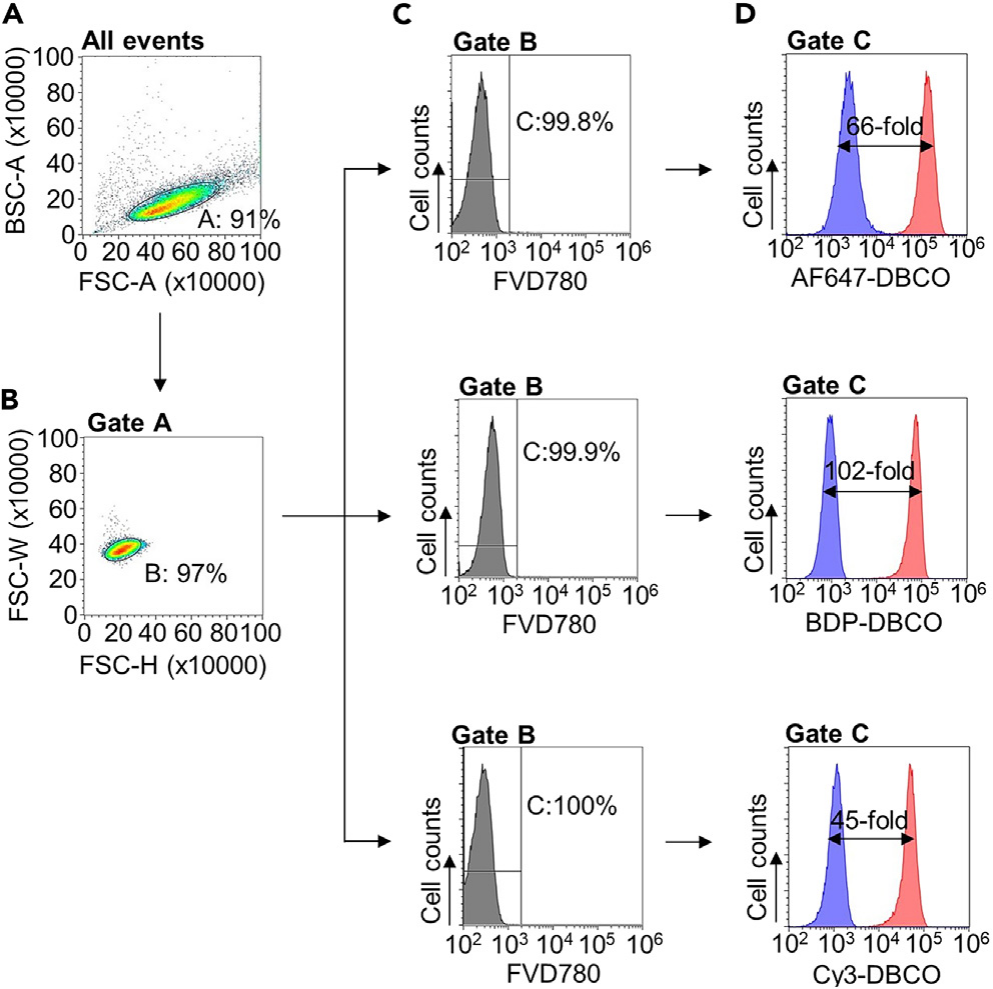
Flow cytometric analysis of labeled PC (A) FSC-A and BSC-A dot plot for exclusion of cell debris. (B) FSC-H and FSC-W dot plot for selection of singlet cells. (C) Histograms showing cell viability of N_3_-Cho-treated cells labelled with AF647-DBCO (top), BDP-DBCO (middle) and Cy3-DBCO (bottom). (D) Histograms showing untreated cells (blue) and N_3_-Cho-treated cells (red) which experienced PC labeling with AF647-DBCO (top), BDP-DBCO (middle) and Cy3-DBCO (bottom).

This section describes the gating strategy for optimal data acquisition (Figure 2).

50. Optimize the flow cytometry instrument setting for optimal data acquisition. If using MA900 (Sony), following parameter can be used as a reference.

**Table:** 

Instrument setting	
Sorting chips	100 μm diameter

**Laser**	

405 nm/488 nm/561 nm/638 nm	On/On/On/On

**Threshold**	

FSC	5%

**Sensor gain**	

FSC	3
BSC	33.5%
FL1: 525/50 for BDP-DBCO	33.0%
FL2: 585/30 for Cy3-DBCO	36.0%
FL10: 665/30 for AF647-DBCO	40.5%
FL12: 785/60 for FVD780	37.0%

51. Scatter density plot (FSC-A vs. BSC-A, all events) (Figure 2A): Set up a plot for FSC vs. BSC area to identify cells of interest.

***Note:*** For K562 cells, typically live cells lie within the range of 20–70 (×10,000) in FSC-A and 10–40 (×10,000) in BSC-A (Gate A). Events with <20 (×10,000) in FSC-A are either debris or dead cells and should be gated out. Any events that lie >70 (×10,000) in FSC-A and >40 (×10,000) in BSC-A are likely non-singlets and should be avoided.

52. Scatter density plot (FSC-H vs. FSC-W, gate A) (Figure 2B): Set up a plot for FSC-H vs. FSC-W to exclude doublet.

***Note:*** Doublet cells can significantly affect your analysis and could lead to inaccurate conclusion. Gate for an area where majority (90%–100%) of events reside (Gate B).

***Optional:*** Live cells can be selected by FVD780-negative staining (Figure 2C).

53. Analysis of fluorescent-labelled PC (Fluorescence vs. Events, gate C) (Figure 2D): set up a histogram plot for each organelle-selectively fluorescent-labelled PC to analyze its distribution.
  a. For analyzing BDP-labeled PC, set up a histogram plot in the green fluorescence channel (FL1 vs. Events for MA900; Figure 2D middle).
  b. For analyzing abundance of AF647-labeled PC, set up a histogram plot in the far-red fluorescence channel (FL10 vs. Events for MA900; Figure 2D top).
  c. For analyzing Cy3-labeled PC, set up a histogram plot in the orange fluorescence channel (FL2 vs. Events for MA900; Figure 2D bottom).

## Expected outcomes

Using the above methods, N_3_-Cho treated cells will display higher fluorescence intensity in labeled PC than untreated cells (Figure 2D). The median fluorescence intensity (MFI) of the PC labeling differs approximately 10- to 100-fold between N_3_-Cho-treated cells and untreated cells (Figure 2D). This method can be combined with CRISPR-Cas9 knockout system or pharmacological experiments to study PC metabolism. Administration of N_3_-Cho into mice should allow PC labeling of cells isolated from tissues and fluids. This method is compatible with conventional cell surface immunofluorescence of live cells using fluorophore-conjugated antibodies for cell lineage identification, but not compatible with intracellular staining.

## Limitations

The protocol shown here is based on floating K562 cells, and therefore appropriate optimization for adherent cells should be required. FBS concentration is critical for avoiding non-specific binding of dyes to cells as well as removing un-reacted dyes. When researchers do not use FBS, this protocol cannot be used. In terms of experimental temperatures, the plasma membrane labeling needs to be conducted below 15°C, in order to minimize endocytosis. The mitochondrial labeling depends on the mitochondrial membrane potential which is maintained at 37°C. Overall, the uncontrolled temperatures potentially affect organelle-selective localization of the dyes and the labeling efficiency in the targeted organelles, which might lead to undesirable results such as nonspecific adsorption of dyes. Thus, adherence to temperature control guidelines in this protocol is critical for achieving organelle selectivity in PC labeling.

## Troubleshooting

### Problem 1

Low or no fluorescence in labeling of PC (related to metabolic incorporation of N3-Cho and organelle-specific labeling of N3-PC).

### Potential solution

This may occur when low or no N_3_-Cho has been incorporated into the cells (step 9), or incorrect concentration of clickable dyes was used (steps 17, 27, or 38). For the former, any of choline remained in the culturing medium should be removed prior to the N_3_-Cho incubation, as choline is a competitive inhibitor of N_3_-Cho. Wash the cells with choline-free medium at least twice, or increase the final concentration of N_3_-Cho. For the later, check the concentration of the clickable dye use (especially for the plasma membrane labeling, which needs higher concentration of the dye than the other labeling). To gain sufficient fluorescence signals in labeling, an optimal ratio of dye amount, cell number and medium volume during click reaction should be determined.

### Problem 2

Non-selective labeling (related to organelle-specific labeling of N3-PC).

### Potential solution

As described in Limitation section, organelle selectivity is sensitive to temperature. Check and control instrumental temperature and incubation time (steps 17, 27, or 38), if unintended staining of dyes is observed.

### Problem 3

Broad or heterogeneous populations in PC labeling (related to flow cytometry).

### Potential solution

This may occur when DBCO reagents and cells are unevenly distributed (steps 17, 27, or 38). Resuspend cells with DBCO working solution and mix well (check the CRITICAL points in organelle-specific labeling of N3-PC).

### Problem 4

Toxicity in mitochondrial washing steps (related to labeling of N3-PC in mitochondria).

### Potential solution

Loss of mitochondrial membrane potential (step 42) promotes removal of unreacted Cy3-DBCO, but the excessive treatment with High K+ washing medium potentially causes cell damage. If obvious toxicity is detected, reduce concentration of mesoxalonitrile 3-chlorophenylhydrazone and valinomycin, or shorten incubation time using High K+ washing medium.

### Problem 5

Altered culture conditions (related to metabolic incorporation of N3-Cho).

### Potential solution

If a medium different from this protocol is used (steps 4–11), efficiency in N_3_-Cho incorporation may be changed. In this case, optimize amount of N3-Cho in cell culture.

## Resource availability

### Lead contact

Further information and requests for resources and reagents should be directed to and will be fulfilled by the lead contact, Itaru Hamachi (ihamachi@sbchem.kyoto-u.ac.jp).

### Materials availability

This study did not generate new unique reagents.

## Data Availability

The previously published article^1^ partly includes the data shown in this protocol. Any additional information required to reanalyze the data reported in this paper is available from the lead contact upon request.
